# Use of Carbon Nanotubes for the Functionalization of Concrete for Sensing Applications

**DOI:** 10.3390/s25123755

**Published:** 2025-06-16

**Authors:** Xiaohui Jia, Anna Lushnikova, Olivier Plé

**Affiliations:** LOCIE, Université Savoie Mont Blanc, CNRS UMR 5271, INES, 73376 Le Bourget du Lac, France; anna.lushnikova@univ-smb.fr (A.L.); olivier.ple@univ-smb.fr (O.P.)

**Keywords:** carbon nanotubes, multifunctional concrete, mechanical properties, electrical properties

## Abstract

This study advances the development of self-sensing concrete through functionalization with carbon nanotubes (CNTs) for structural health monitoring. Through experimental analyses, it relies on its dual responsiveness to mechanical and thermal stimuli. Three-point bending and thermal tests were systematically conducted on concrete samples with CNT concentrations ranging from 0 to 0.05 wt.% of cement, evaluated at 7- and 28-day curing periods. Mechanical testing demonstrated curing-dependent behavior: At 7 days, mechanical strength and electrical current response exhibited pronounced variability across CNTs loadings, with optimal balance achieved at 0.01% CNTs. At 28 days, the tests show that the mechanical properties are relatively stabilized, reaching the highest value at 0.006 wt.% CNTs and achieving the best electrical sensitivity at 0.01 wt.% CNTs. The thermal experiments revealed faster current modulation in the 7-day samples than in the 28-day counterparts, with intermediate CNT concentrations (e.g., 0.01 wt.%) showing a more sensitive response. The sensitivity was analyzed for both mechanical and thermal changes to further evaluate the feasibility of using CNT-reinforced concrete as a sensor material. Conductivity measurements on fully cured samples indicated that all samples exhibited electrical conductivities in the 10^−4^ S/m range, suggesting semiconductive behavior, while 0.006 wt.% CNTs yielded the highest conductivity. Higher CNT content did not further improve conductivity, likely due to agglomeration disrupting the network. These findings confirm CNT-modified concrete’s dual electromechanical and thermal responsiveness and support its potential as a multifunctional sensing material.

## 1. Introduction

As the most used building material, concrete is a composite material made of cement, water, and aggregates. It is an indispensable material in modern construction, valued for its high strength, affordability, durability, and versatility [1,2,3,4]. However, nowadays, the use of concrete is criticized because of its high carbon footprint. According to statistics by the Netherlands Environmental Assessment Agency, carbon emissions from cement production account for approximately 8% of global carbon emissions [5]. Additionally, the United Nations Environment Programme highlighted in its Emissions Gap Report 2024 that carbon emissions from the buildings sector alone account for 6% of global carbon emissions, excluding emissions from cement production and carbonation [6]. In response to this debate, many researchers have contributed to improving concrete’s mechanical performance, durability, and environmental sustainability, aiming to make concrete more environmentally and functionally appealing. The incorporation of nanomaterials into concrete has become a popular research focus, driven by advancements in nanotechnology [7,8,9,10]. Commonly used nanomaterials include graphene, carbon fiber, carbon nanotubes (CNTs), nano-TiO_2_, and nickel particles [4,11].

Among all nano-enhanced materials, multi-walled carbon nanotubes (MWCNTs) are chosen as reinforcing fillers in cementitious composites because of their unique structural advantages and multifunctional characteristics, such as their excellent mechanical, electrical, and thermal properties [12]. MWCNTs demonstrate superior load transfer efficiency compared to conventional reinforcement materials because of their high aspect ratio and remarkable tensile strength [13]. Furthermore, the tubular structure provides additional nucleation sites for cement hydration products, accelerating the formation of C-S-H gel and densifying the matrix microstructure [14].

Numerous studies have incorporated varying proportions of MWCNTs into concrete to investigate their effects on enhancing its mechanical and functional performance. Among them are studies focusing on self-sensing concrete reinforced with carbon nanotubes. Xu et al. [15] investigated the mechanical properties of cement pastes reinforced with MWCNTs at dosages ranging from 0.025 wt.% to 0.1 wt.% and concluded that the compressive strength and flexural strength were improved most with the 0.1 wt.% MWCNTs contents. Kanagaraj et al. [16] investigated the effects of MWCNTs (0.02% to 0.16%) on geopolymer concrete, focusing on compressive strength and load-deflection behavior. The results showed that 0.12% MWCNT content achieved the highest compressive strength, while 0.06% provided the optimal Flexural Toughness Factor. Yahyaee et al. [17] studied the use of MWCNTs in porous concrete and found that as the percentage of MWCNTs increased from 0.01% to 0.02%, the compressive strength of the concrete improved accordingly. Shahzad et al. [18] investigated the effects of CNT content (0%, 0.5%, 0.75%, and 1%) on electrical and mechanical properties, concluding that a minimum CNT content of 0.75% is required to achieve significant self-sensing sensitivity without compromising the mechanical properties of the cementitious material. Lee et al. [19] explored the self-sensing capability of ultra-high performance fiber-reinforced concrete incorporating 0.1 wt% and 0.5 wt% MWCNTs. The developed sensor demonstrated excellent strain sensitivity, and beam tests further confirmed its effectiveness in damage detection. Siahkouhi et al. [20] investigated the self-sensing behavior of concrete with three different CNT contents (0.5%, 0.75%, and 1.0%) from the perspective of rail health monitoring. The experimental results demonstrated that carbon nanotubes exhibit sensitivity to applied loads, with 0.5% CNT content identified as the optimal proportion.

However, the optimal content of CNTs remains inconclusive, as factors such as CNT type, fabrication method, dispersion technique, and experimental approach can influence the results. To further validate this, the present study builds upon previous research conducted within our group by using a selected proportion for comparative verification. Additionally, based on findings from other studies, several variable groups were added within a reasonable range for further exploration, with CNT content varying up to 0.05 wt.% relative to the mass of cement.

This research fabricated concrete samples with varying proportions of MWCNTs and conducted three-point bending and thermal tests after 7 and 28 days of curing. The mechanical and electrical performance of the material is evaluated under loading between 2 and 3 kN for the three-point bending tests and a heat flux of 200 W/m^2^ for thermal tests. The electrical conductivity of concrete samples cured for 6 months was measured using the two-electrode method to evaluate the effect of CNTs on the electrical conductivity of concrete. Additionally, an advanced LabVIEW-Picoammeter system has been developed to record real-time current variations during testing. As a result, the electrical response of MWCNT-reinforced cementitious composites under mechanical deformation and temperature changes was obtained.

This study is one of several aimed at adding functionalities to concrete, in particular its ability to provide information on its mechanical state, to make it a sensor. This sensor could be used in reinforced concrete structures to ensure maintenance and plan renovations. Meanwhile, it provides technical support for intelligent monitoring in the construction industry and shows significant potential for the development of smart structural health monitoring systems for the next-generation infrastructure.

## 2. Materials and Methods

### 2.1. Materials and Sample Preparation

Graphistrength^®^ CW2-45 [21] is a solid masterbatch developed by ARKEMA, composed of 45 wt.% multi-walled carbon nanotubes (MWCNTs) and 55 wt.% carboxymethyl cellulose (CMC). It is designed for easy dispersion in liquid thermoset or water-soluble formulations. The maximum CNT content in this study was set at 0.5 wt.%, with additional concentrations of 0 wt.%, 0.002 wt.%, 0.006 wt.%, 0.01 wt.%, 0.018 wt.%, and 0.025 wt.%. This range was selected based on previous work using the same CNT Masterbatch from Arkema to assess the influence of both minimal and moderate CNT dosages on material properties [22,23]. The concrete samples used for testing were prepared following the EN 196-1 standard. The components and their respective quantities are detailed in Table 1.

**Figure 1:**
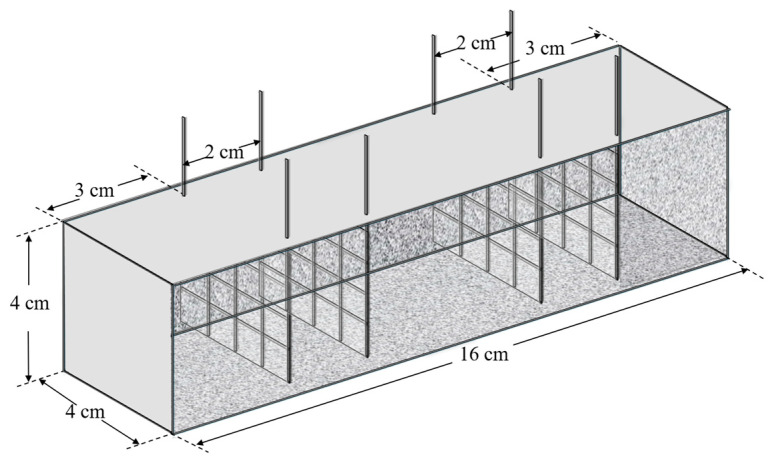
Inox grid placement methods.

To measure the electrical conductivity and related properties of the concrete samples (4 cm × 4 cm × 16 cm), conductive inox grids were embedded within the specimens, as illustrated in Figure 1. The grids were positioned 3 cm from the sample edges, with a 2 cm gap between the two grids on the same side. This configuration enables the assessment of the electrical properties across the entire sample and facilitates the testing of smaller sections after mechanical experiments. This approach maximizes material utilization and minimizes waste.

**Figure 2:**
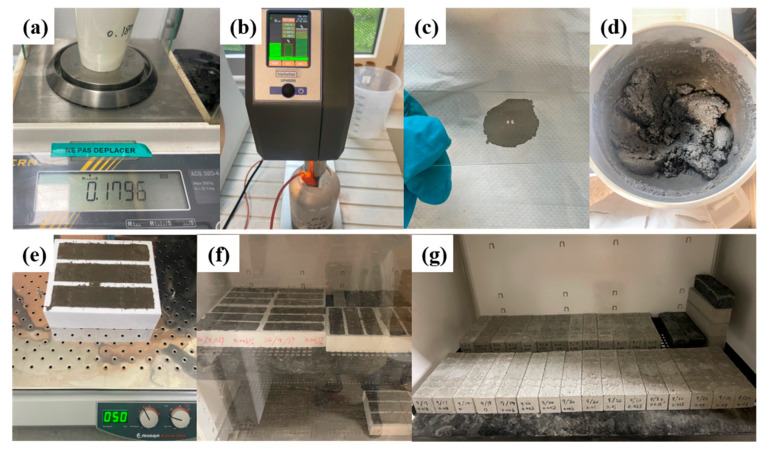
Samples preparation procedures: (**a**) Weighing Materials; (**b**) Dispersal of CNT; (**c**) Assessing Dispersion Uniformity; (**d**) Preparing Cement Paste; (**e**) Casting and Vibrating; (**f**) Demolding; (**g**) Curing.

The experimental procedure shown in Figure 2 involves the following steps: (a) Weighing Materials: The materials are weighed using precise balances. CNT batches are measured with a balance that has 0.1 mg precision due to their low weight, while other materials such as water, sand, and cement are weighed using a balance with 0.1 g precision. (b) Dispersing CNTs: The particles are first placed in hot water (approximately 70 °C) and manually stirred with a glass rod until dissolved, with no visible undissolved particles remaining. Then the dispersion is processed using a Hielscher UP400St ultrasonic processor for 5 min with an applied power of 40 W, which is within the effective range reported in the literature [24] for CNTs dispersion. The ultrasonic processor is equipped with a temperature sensor to measure the temperature of the solution, ensuring the temperature stays below 90 °C to prevent the evaporation of water, which could influence the experimental results. (c) Assessing Dispersion Uniformity: To evaluate the dispersion uniformity, a drop of the solution is placed on a blank glass slide for initial observation; uniform black coloration without visible aggregates suggests reasonably good dispersion. (d) Preparing Cement Paste: A mixer is used to prepare the cement paste according to the EN 196-1 standard. Notably, the water in the standard procedure was substituted with the solution obtained from step (b) to ensure uniform integration of the nanotubes into the cement matrix. (e) Casting and Vibrating: The well-mixed mortar is poured into molds, each capable of producing three samples with dimensions of 4 cm × 4 cm × 16 cm. The filled molds are placed on a vibrating table and vibrated at speed level 3 for 5 min to eliminate air bubbles, resulting in denser samples. (f) Demolding: The samples are placed in curing rooms with controlled humidity for 3 days before being removed from the molds. (g) Curing: After demolding, the samples are returned to the curing room for continued curing until further testing.

### 2.2. Conductivity Test Setup

**Figure 3:**
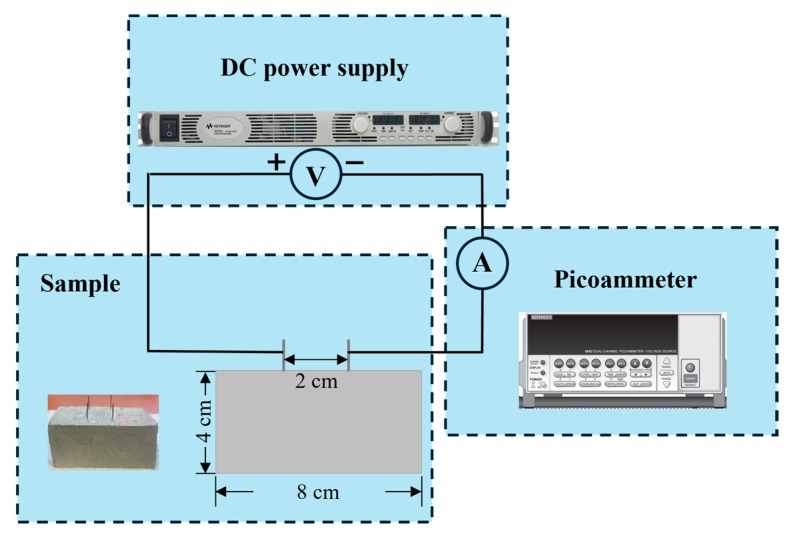
Electrical conductivity test setup.

To investigate the effect of CNT content on the electrical conductivity of concrete samples, 7 samples with varying CNT contents (0 wt.%, 0.002 wt.%, 0.006 wt.%, 0.01 wt.%, 0.018 wt.%, 0.025 wt.%, and 0.05 wt.%) were tested using the setup shown in Figure 3. The size of the sample is 4 cm × 4 cm × 8 cm, which is half the dimensions of the samples described in Section 2.1, with two stainless steel mesh grids embedded and used as electrodes. A Keysight (formerly Agilent) N5768A programmable DC power supply (0–80 V range, 0.01 V accuracy) was used to apply voltage, while a Keithley Model 6482 Dual-Channel Picoammeter was used to measure the current passing through the samples. According to the instrument specifications, the accuracy depends on the selected range. For example, on the 2 nA range, the accuracy is ±(1.00% of reading + 2 pA). The measurements followed a six-point method, in which six different voltages (0 V, 2 V, 4 V, 6 V, 8 V, and 10 V) were sequentially applied, and the corresponding current at each voltage level was recorded. The resistance *R* of the specimen was obtained from the slope of the resulting voltage–current fitting curve.

To minimize temperature change and potential electrode polarization effect caused by continuous power supply, the power was disconnected immediately after each current recording. The voltage was then adjusted before reconnecting the circuit to conduct the next measurement. This procedure helps reduce errors introduced by thermal or interfacial effects. Based on the conduction distance *L* = 0.02 m and the sample cross-sectional area *S* = 0.0016 m^2^, the resistivity *ρ* was calculated as:

(1) $$\rho =R\times \frac{S}{L},$$

The electrical conductivity *σ* was then obtained as the reciprocal of resistivity:

(2) $$\sigma =\frac{1}{\rho },$$

To reduce measurement errors, each sample batch was measured twice, and the average resistance value was used in subsequent calculations. Since moisture content has a significant influence on the electrical conductivity value, the specimens used in this test were fully cured for six months to reduce the impact of water. It should be noted that the aim of this study was not to determine precise conductivity values, but rather to compare the relative influence of CNT content on electrical conductivity. Therefore, factors such as temperature, moisture content, and sample size were kept constant and not investigated further.

### 2.3. Three-Point Bending Test Setup

**Figure 4:**
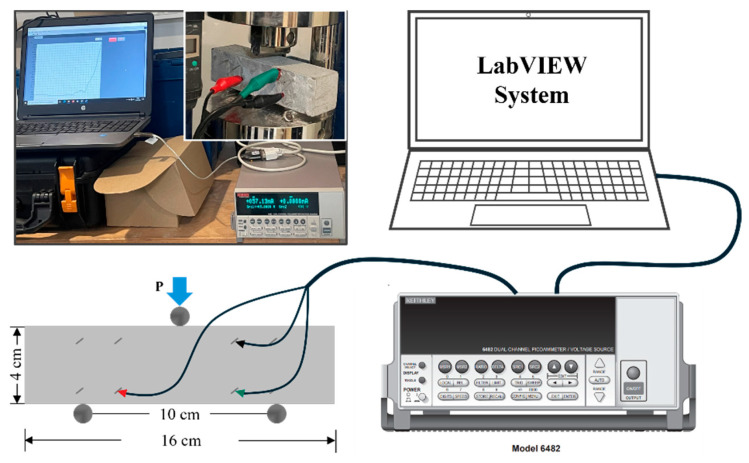
Three-point bending test setup with LabVIEW-Picoammeter system.

To verify the effect of CNT content on the mechanical properties of the concrete samples and their ability to sense mechanical deformations, concrete samples with varying CNT contents (0 wt.%, 0.002 wt.%, 0.006 wt.%, 0.01 wt.%, 0.018 wt.%, 0.025 wt.%, and 0.05 wt.%) were subjected to three-point bending tests after 7 and 28 days of curing. The tests were performed using a Controlab hydraulic press with a loading rate of 0.050 kN/s and an initial load of 1.0 kN. During the tests, time, corresponding load, maximum load, and maximum strength were recorded. A triaxial cable was used to connect the stainless-steel grid embedded in the sample to the input channel of the Picoammeter. The current flowing between the grids was continuously monitored and recorded in real-time at 1 s intervals. Data acquisition was initiated by clicking the start button on the LabVIEW terminal simultaneously with the commencement of the bending test. The experimental setup is shown in Figure 4. To minimize experimental errors, each test (for a given CNT content and curing duration) was repeated three times. For data visualization, the middle value was selected to draw the curves. The result with a significant deviation from the other two was considered an outlier. The average of the remaining two values was calculated to plot the maximum load figure. Additionally, the smoothness of the current curve was considered to ensure that the selected dataset accurately represents the typical behavior of the sample.

### 2.4. Thermal Test Setup

**Figure 5:**
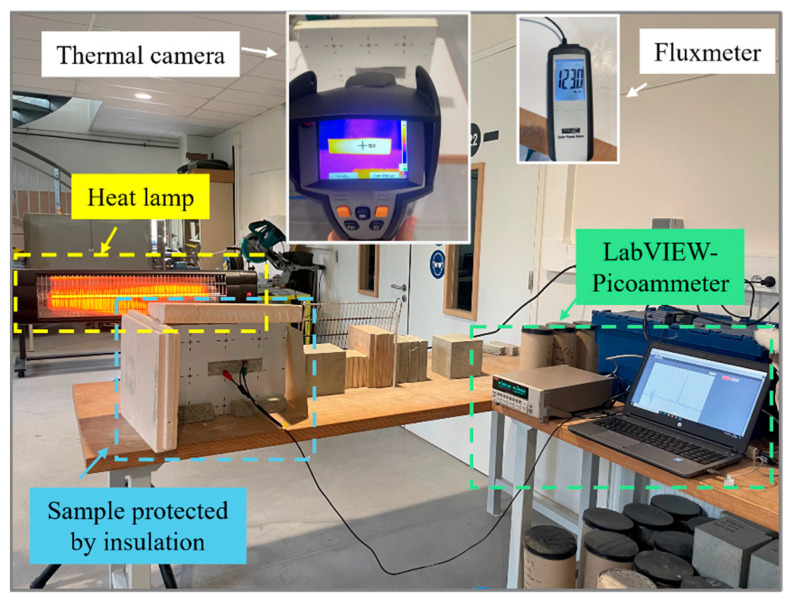
Thermal test setup with LabVIEW-Picoammeter system.

To evaluate the influence of CNT content on the temperature-sensing capability of concrete samples, thermal tests were conducted on specimens with varying CNT concentrations (0 wt.%, 0.002 wt.%, 0.006 wt.%, 0.01 wt.%, 0.018 wt.%, 0.025 wt.%, and 0.05 wt.%). The samples were heated to 50 °C using a lamp, while the induced current was continuously recorded via the inox grid embedded in the concrete, which was connected to a LabVIEW-Picoammeter system. Thermal insulation was applied to protect the wires from overheating and prevent measurement interference. A thermal camera was used to monitor surface temperature throughout the experiment, and a fluxmeter was used to measure and ensure a consistent heat flux on the sample surface. The experiment setup is shown in Figure 5, and the ambient temperature at which this experiment was performed was 23 °C room temperature. To reduce the error, the same test was performed twice, and the data that best represents the typical behavior was chosen to plot the curves.

## 3. Results

### 3.1. Conductivity Test

**Figure 6:**
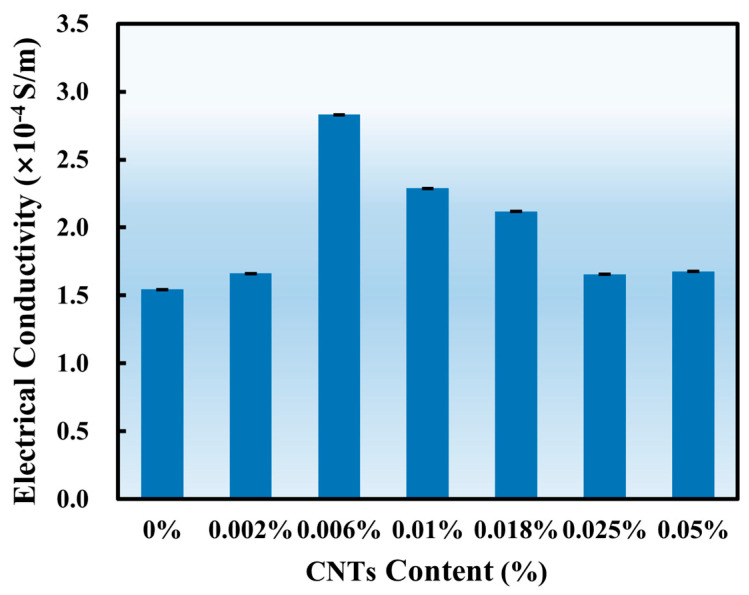
Conductivity values of samples with different CNTs contents.

Several experiments were conducted on mature concrete samples with varying CNT contents to obtain electrical conductivity. The results from measurement and calculation are shown in Table 2. As described in Section 2.2, to minimize experimental errors, each test was conducted twice. In each group, a six-point measurement method was employed, and the resistance was determined by fitting curves of different voltage (U) and current (I) values. Here, R_1_ represents the resistance obtained from the first measurement, while R_2_ corresponds to the second. For each fitting curve, the R^2^ value exceeded 0.985, indicating the reliability of the data. Accordingly, *ρ*_1_ and *ρ*_2_ represent the resistivity calculated from the first and second measurements, respectively, while *σ*_1_ and *σ*_2_ denote the corresponding electrical conductivities. The final electrical conductivity of the sample, *σ*, is expressed as the average of *σ*_1_ and *σ*_2_, which are shown in Figure 6.

As illustrated in Figure 6, all samples, including the control group with 0% CNTs, exhibit electrical conductivities in the order of 10^−4^ S/m, which shows semiconductor properties [25]. At first glance, this same scale phenomenon might suggest that CNTs have a limited effect in strengthening the semiconducting behavior of concrete. However, testing conditions should not be ignored: the samples have undergone six months of long-term curing, which means they have low moisture content. As a result, the ionic conduction, which is the primary conduction mechanism in moist cementitious materials, is minimized [26]. The remaining conduction is largely determined by the conductive pathways caused by CNTs. Under this premise, even a minor change in conductivity can prove the impact of CNTs embedded in concrete. It can be seen that the samples with CNTs show an improvement in conductivity, particularly at 0.006%, where the highest value is observed. This indicates that low CNT contents promote the formation of a more continuous and efficient conductive network. Beyond the optimal point (0.006%), further addition leads to a decline in performance, likely due to CNT agglomeration, which interrupts the conductive network. This phenomenon proves that the improvement in conductivity with CNTs is realistic and is sensitive to dispersion quality and dosage control.

The presence of semiconductive behavior in the control sample does not negate the contribution of CNTs; it sets a baseline under dry conditions, under which the enhancement due to CNTs can be quantitatively evaluated. Araújo et al. [27] found that shorter electrode spacing leads to lower measured resistivity, which corresponds to higher electrical conductivity. Dehghanpour et al. [28] reported that sample size significantly influences the electrical resistivity of cube-shaped specimens, with smaller samples exhibiting lower electrical resistivity and thus higher electrical conductivity. In the present study, the specimens used have dimensions of 4 cm × 4 cm × 8 cm, with an electrode spacing of 2 cm. This small sample size and short electrode distance help explain why the control group also exhibited relatively high electrical conductivity.

### 3.2. Three-Point Bending Test

#### 3.2.1. 7-Day Concrete

**Figure 7:**
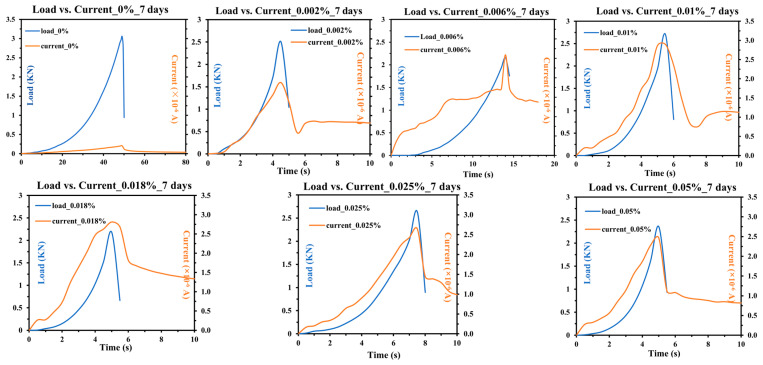
Load vs. current variation over time of 7-days concrete (axes of the same scale are partially omitted for clarity).

Three-point bending tests were conducted on concrete samples with different CNT contents at an age of 7 days. The experimental results depicting the variations in load and current over time are shown in Figure 7. The load–time curves (plotted in blue) illustrate the mechanical response of the samples, while the current–time curves (plotted in orange) reflect the real-time electrical behavior during mechanical deformation.

**Figure 8:**
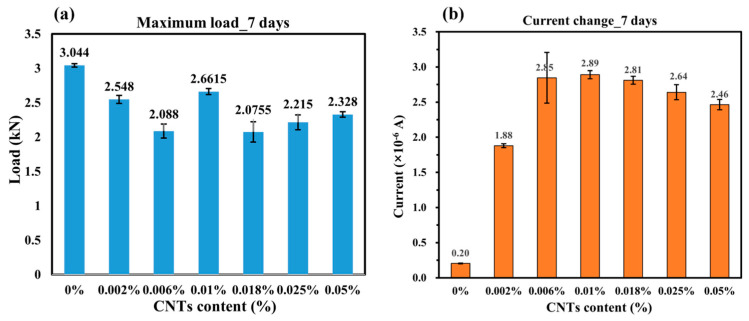
(**a**) Maximum load of 7-days concrete; (**b**) Current change of 7-days concrete.

As the CNT content increases, the peak load sustained by the samples exhibits a slight variation. The control sample (0% CNT) demonstrates a distinct load–time curve with a gradual increase until failure, whereas samples incorporating CNTs exhibit different peak loads and failure behaviors. This variation suggests that CNT inclusion influences the mechanical performance of concrete, potentially altering crack propagation mechanisms and structural integrity under bending stress. The coordinate axes in the figures are kept consistent (except for the control sample), allowing direct comparison of peak values. Figure 8a exhibits that, as the CNT content increases, the trend initially declines before recovering; the overall peak load values remain lower than those of the control sample. It is observed that the control sample exhibits the highest mechanical performance, with a maximum load of 3.044 kN, then the second highest value, which is obtained when the CNT content is 0.01 wt.%, which is 2.6615 kN.

The current–time curves reveal a notable correlation between mechanical loading and electrical response. For all CNT-doped samples, the current initially increases with loading and reaches its peak at the maximum load that corresponds to the highest mechanical deformation. This strong consistency between mechanical deformation and electrical responses suggests that a piezoresistive behavior exists in the CNT-reinforced composites. When the sample is subjected to mechanical loading, microscopic deformation causes an increase in the number of contact points between CNTs and the cement matrix. It leads to the reduction in contact resistance, thereby enhancing electron transport pathways within the CNT network, and facilitating more efficient electron transport [27]. Additionally, the deformation of CNTs, including bending or stretching, may modify their electronic structure and thus further influence their conductivity [29].

After the sample reaches peak load and begins to fail, the current does not exhibit an abrupt drop. On the contrary, it declines gradually, indicating that although cracks have formed, the sample does not fully break apart, preserving a continuous electrical pathway. This behavior suggests that CNT-enhanced concrete retains a degree of conductivity even after structural damage, which is advantageous for self-sensing applications. The current values for CNT-doped samples are significantly higher than the control sample. This phenomenon can be explained through the lens of Yan’s research [13], which reveals that CNTs permeate the void spaces and pore networks within the concrete matrix, effectively interconnecting disparate components of the composite. When subjected to mechanical stress, these CNTs function as nanoscale bridges between fractured regions, thereby establishing continuous conductive pathways through electron tunneling and percolation mechanisms. However, like the mechanical performance, the electrical response does not exhibit a strictly increasing trend but instead fluctuates with different CNT concentrations. As shown in Figure 8b, as the CNT content increases, the current change initially increases and reaches its highest value at 0.01% CNT content, which is 2.89 × 10^−6^ A, then the current change shows a decreasing trend.

With increasing CNT content, both mechanical performance and electrical monitoring capability exhibit an inconsistent trend. However, beyond a certain concentration, the enhancement in mechanical strength may plateau, while excessive CNT concentrations could lead to the agglomeration of nanotubes that negatively impact the uniformity of the conductive network. Identifying an optimal CNT concentration that balances both mechanical integrity and electromechanical sensing performance is thus crucial. Based on the combined analysis of mechanical and electrical properties, the sample with 0.01% CNT content demonstrates the most sensitive electrical response while maintaining mechanical performance without significant degradation. This suggests that 0.01% CNT is an optimal concentration of 7-day concrete for achieving both structural integrity and effective self-sensing capability.

#### 3.2.2. 28-Day Concrete

**Figure 9:**
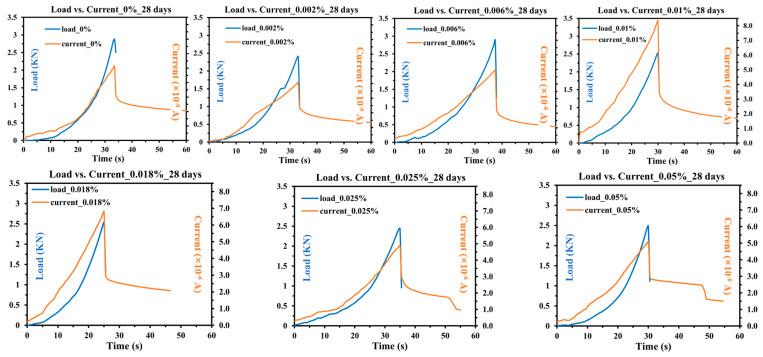
Load vs. current variation over time of 28-days concrete (axes of the same scale are partially omitted for clarity).

The results of three-point bending tests conducted on 28-day cured concrete samples with varying CNT content are shown in Figure 9. The blue curves represent load varying with time, which illustrates the mechanical response of the samples, while the orange color represents the current–time curve, which reflects the real-time electrical behavior during mechanical deformation. The experimental results reveal notable trends in both mechanical and electrical responses. The load–time and current–time curves exhibit a high degree of consistency, with the peak current values occurring approximately at the same time as the maximum applied load. This indicates a strong correlation between mechanical deformation and electrical signal variation. This consistency suggests that the conductive pathways formed by the CNTs within the concrete matrix respond dynamically to mechanical stress. After the sample begins to fail, the load decreases sharply, whereas the current exhibits a more gradual decline. This reflects that conductive pathways are still partially maintained even after crack formation. As a consequence, this suggests that while microcracks disrupt the network, complete disconnection does not occur immediately. This is consistent with the experimental results of 7-day concrete.

**Figure 10:**
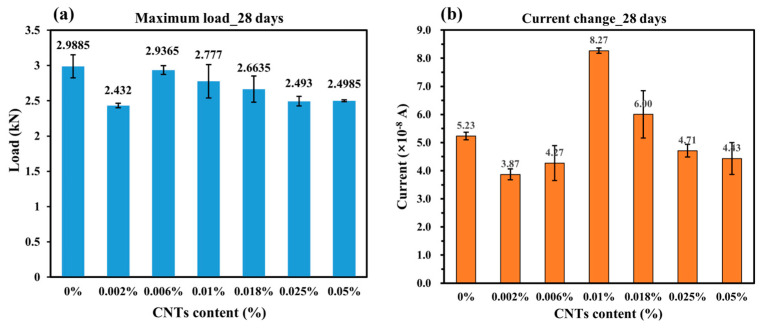
(**a**) Maximum load of 28-days concrete; (**b**) Current change of 28-days concrete.

As shown in Figure 10a, the mechanical performance of 28-day cured concrete remains relatively stable, with only minor variations in peak load across different CNT contents. Analyzing the peak values across different CNT concentrations, the highest load-bearing capacity is observed at 0 wt.% CNT content, reaching 2.9885 kN, followed by a high value of 2.9365 kN obtained at 0.006 wt.%; this CNT content aligns with findings from [22], which reported the same results for CNT-reinforced cementitious composites. This difference in optimal CNT content between the samples of 7 days and 28 days can be attributed to the evolution of the cement matrix. At 7 days, due to the incomplete state of the cement hydration process, the matrix remains relatively porous and has higher humidity, allowing CNTs to keep better contact with the matrix. Also, the humidity influences the sensing property of CNTs [30]. Thus, in this early stage, a higher CNT content dominantly enhances concrete’s mechanical properties by crack-bridging and pore-filling [31]. However, at 28 days, the matrix becomes drier and denser due to the hydration process, which may reduce the effective contact area between CNTs and the matrix. In contrast, a lower CNT content at this stage tends to be more uniformly distributed, leading to a more favorable effect on the mechanical properties.

In contrast, the current response exhibits a different trend, with peak values appearing at intermediate CNT concentrations, as shown in Figure 10b. The maximum current variation is observed at 0.01 wt.% CNT, reaching 8.27 × 10^−8^ A, suggesting that this concentration achieves the best balance between electrical sensitivity and mechanical integrity. This also indicates that while a small CNT addition may reinforce mechanical strength, excessive CNT concentration does not further enhance the load-bearing capacity, due to potential agglomeration and defects within the matrix.

Determining an exact optimal CNT concentration is challenging, as different performance indicators peak at different levels. However, within the range of 0.006 wt.% to 0.018 wt.% CNT, the samples exhibit stable mechanical performance and reach the highest value at 0.006 wt.% CNT, while achieving the best electrical sensitivity at 0.01 wt.% CNT. This suggests that for applications that prioritize self-sensing capabilities without compromising structural integrity, a CNT concentration at 0.01 wt.% is preferable.

Comparing these results with the 7-day curing data, the 28-day cured samples exhibit greater stability and higher overall mechanical strength. However, the electrical response is significantly reduced. This can be attributed to the nature of concrete hydration. Over time, the hydration process leads to a denser matrix with lower porosity and reduced free water content. The decline in the electrical response reflects that ionic conduction plays a significant role in the early-age electrical behavior of CNT-enhanced concrete [32]. As the hydration progresses and the free ions are consumed or immobilized, the ionic conductivity decreases. This leads to lower overall current values at 28 days. This indicates that while CNTs contribute to electrical conductivity, their effect is modulated by the evolving microstructure of the cementitious matrix. Understanding this interaction is crucial for optimizing CNT content to achieve both reliable mechanical performance and effective self-sensing capabilities over time.

### 3.3. Thermal Test

#### 3.3.1. 7-Day Concrete

**Figure 11:**
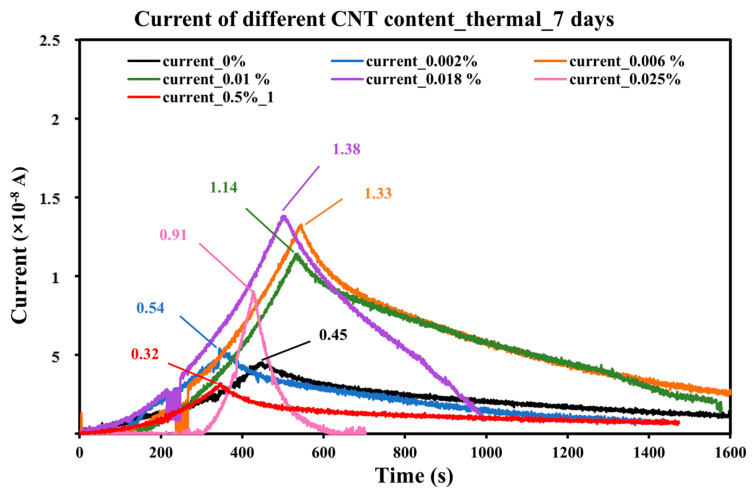
Current vs. time of 7-days concrete with different CNT contents.

Thermal tests were conducted on concrete samples with different CNT contents at an age of 7 days. The current vs. time curves are plotted in Figure 11 based on experimental results, which illustrate the electrical response during the heating procedure, and different colors are used for the curves to distinguish between different CNT contents. The experimental parameters and corresponding current responses of CNT-modified concrete samples are listed in Table 3. The table includes the CNT content, initial and final temperatures (T_0_ and T_1_), applied heat flux, heating duration, maximum detected current, and the total energy received by the sample surface.

It can be seen from Figure 11, as the heating time increases, the current detected increases accordingly. When the sample surface reaches 50 °C, the current response peaks. Subsequently, the heat source is turned off, and the sample begins to cool under ambient conditions. As the temperature drops, the current decreases. The recording process is terminated when the sample cools to 30 °C, by which point the current stabilizes at a baseline level.

It can also be observed from the figure that the current response patterns vary with different CNT contents. Samples with higher CNT contents generally reach higher current peaks, indicating a stronger thermal–electrical coupling effect. However, the peak value starts to decrease when the CNT content is higher than 0.018 wt.%. Among all the samples, the sample with a CNT content of 0.05% has the lowest peak current, which is lower than the control sample without CNT. The samples with CNT contents of 0.006 wt.%, 0.01 wt.%, and 0.018% show minimal differences in both the time required to reach the peak current and the peak current value itself. Considering the previous current response results obtained from mechanical tests, subsequent experiments will prioritize testing within this concentration range.

Referring to Table 3, the sample with CNT content of 0.002 wt.% of cement requires the least amount of time to reach 50 °C, achieving its current peak in approximately 344 s; following it is the sample of CNT content of 0.05 wt.% of cement, which requires 345 s to reach the peak. In contrast, the sample with 0.018 wt.% CNT content exhibits the highest current peak of 1.38 × 10^−8^ A, highlighting its enhanced conductivity due to the denser CNT network. Meanwhile, the samples with 0.006% and 0.01% CNT content also show high responses, both achieving their peak on a scale of 10^−8^ A. 

From the perspective of the thermal energy collected by the sample, the peak current response can be directly related to the total thermal energy delivered to the sample surface. Since the heat lamp used in the experiment has a large surface area, it provides a uniform heat flux over the sample surface, as confirmed by the flux meter measurements. It is known that heat flux is a flow of energy per unit area per unit time [33]. An equation can be obtained by backtracking from the definition to calculate the total energy gained at the sample surface, where possible energy losses are ignored since all samples were performed under the same experimental setup. Thus, the total thermal energy absorbed by the sample can be calculated using the following equation:

Q = Φ × A × t,(3)

where Q is the total thermal energy absorbed (in joules), Φ is the absorbed heat flux (in W/m^2^), A is the sample surface area (0.0064 m^2^), and t is the heating time (in seconds).

This equation illustrates that the energy absorbed by the sample is directly proportional to the heating duration and the applied heat flux. This indicates that when the heat flux is constant, the maximum current value is not only related to the CNT content but also to the heating duration. Also, the heat flux delivered to the sample surface accumulates, leading to a progressive rise in the internal temperature of the material. This temperature increase induces charge-carrier excitation within the CNT network, enhancing electron mobility and resulting in a gradual increase in the measured current. In addition, the time required to reach 50 °C provides a more intuitive reflection of the sample’s sensitivity to temperature changes.

#### 3.3.2. 28-Day Concrete

**Figure 12:**
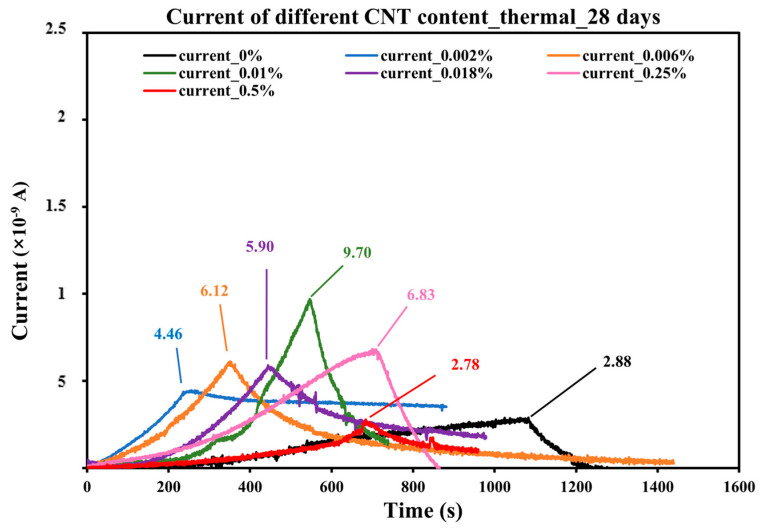
Current vs. time of 28-days concrete with different CNT contents.

The same thermal experiments were conducted on concrete samples at an age of 28 days. The current vs. time curves are plotted in Figure 12, which illustrates the electrical response during the heating procedure of different CNT contents. The corresponding parameters and results of CNT-modified concrete samples are listed in Table 4.

The current response patterns in the 28-day samples exhibit similar trends to those in the 7-day samples. As the heating time increases, the current detected increases accordingly until it reaches its peak at 50 °C. Then, since the heat source was turned off, it started decreasing. The recording process is terminated when the sample cools to 30 °C.

Since the same scales are used for both axes, it is visually evident that the current peaks at 28 days are significantly lower than those at 7 days. This difference may stem from the fact that the 7-day samples had not yet completed hydration, leading to higher internal water content. The presence of water promotes ionic conduction, enhancing the current response. As hydration continues over time, a decrease in free water content likely reduces the sample’s conductivity, resulting in a less pronounced current signal. In contrast to the 7-day samples, where current peaks appeared within a certain range, the 28-day samples showed more varied peak values. Notably, the sample with 0.01% CNT content exhibited the highest current peak, reaching 9.70 × 10^−9^ A.

According to Table 4, the time required for the samples to reach 50 °C varies significantly, indicating that prolonged curing time influences not only the maximum current but also the rate of current buildup. Moreover, the current peak values no longer correlate directly with the total absorbed energy. The control sample with 0% CNT content exhibited the longest heating duration and the highest total energy absorption. However, it generated the lowest current response, highlighting the critical role of CNTs in enhancing the material’s thermal–electrical sensitivity.

This phenomenon can be explained by the role of CNTs in forming conductive networks within the concrete matrix. In CNT-doped samples, as the temperature rises, thermal expansion may enhance contact between CNTs or promote electron hopping, leading to a stronger current response [34]. In contrast, the control sample lacks these conductive pathways, meaning ion migration through the pore solution becomes the primary conduction mechanism, which is inherently less efficient, especially as the sample loses moisture over time.

Furthermore, the time to peak current can also be interpreted as an indicator of thermal sensitivity. Samples with intermediate CNT content (e.g., 0.01%) reached 50 °C faster and produced a higher current peak, suggesting an optimal balance where CNT networks efficiently conduct charge without excessive agglomeration, which could otherwise hinder conductivity.

### 3.4. Mechanical and Thermal Sensitivity

**Figure 13:**
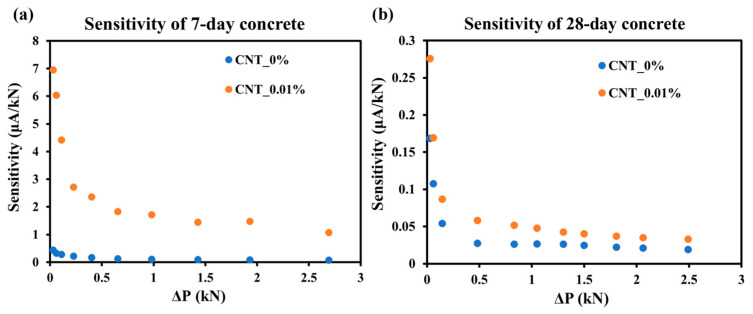
Sensitivity to load of (**a**) 7-day concrete; and (**b**) 28-day concrete.

Sensor sensitivity is generally defined as the change in output signal per unit change in input, reflecting the sensor’s responsiveness [35]. To evaluate the electrical sensitivity in response to a load of different-CNT-content concrete samples, concrete without CNT (CNT_0%) and concrete with the optimal content of 0.01% (CNT_0.01%) were selected to represent the sensitivity. The mechanical sensitivity was calculated as the ratio of electrical current to applied load change (ΔP) based on the data points recorded under the same loading conditions. The resulting sensitivities were plotted as shown in Figure 13.

Figure 13a,b illustrate the sensitivity–load relationships for 7-day and 28-day cured samples, respectively. A significant difference can be observed between the two groups: the 7-day cured concrete specimens exhibited higher sensitivity compared to the 28-day specimens. The 7-day cured concrete specimens exhibited sensitivity below 7 μA/kN, while the 28-day samples demonstrated significantly lower sensitivity values under 0.3 μA/kN. CNTs demonstrate more pronounced enhancement effects on the sensitivity for 7-day samples, which is consistent with previous tests. For both curing ages, the CNT_0.01% samples exhibited consistently higher sensitivity values than the CNT_0% samples, particularly in the low-load region (<1 kN), where the sensitivity of the current response was more pronounced. As the load increased, sensitivity decreased accordingly and gradually stabilized for both groups. This is attributed to the fact that as the load is applied, the sample is on the verge of failure and the internal conductive network is destroyed. These results confirm the enhanced electromechanical response of CNT-reinforced concrete, supporting its potential as a self-sensing material for structural health monitoring applications in damage detection.

The temperature sensitivity of the current response in concrete samples with different CNT contents was analyzed using the same methodology as for mechanical sensitivity. In contrast to mechanical loading starts from zero, the temperature increases from room temperature (23 °C) to 50 °C, corresponding to a ΔT of 0–27 °C. The thermal sensitivity was consequently defined as the ratio between the measured current and the temperature change, expressed in nA/°C.

**Figure 14:**
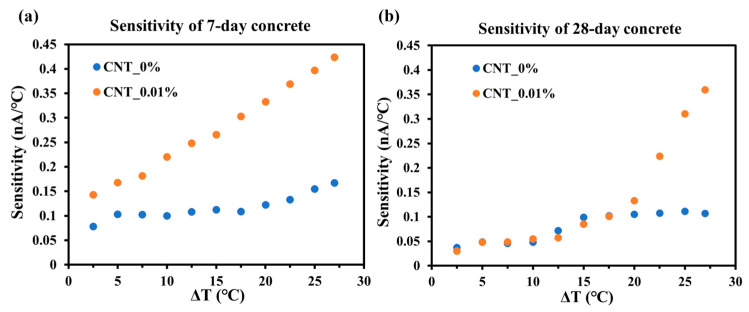
Sensitivity to temperature of (**a**) 7-day concrete; and (**b**) 28-day concrete.

Figure 14a,b present the temperature sensitivity of 7-day and 28-day cured concrete specimens (CNT_0% and CNT_0.01%), respectively. In general, the 7-day samples exhibited greater sensitivity to temperature changes than the 28-day samples, aligning with previous results. As shown in Figure 14a, the sensitivity of both CNT_0% and CNT_0.01% samples increase with increasing temperature. The concrete sample without CNT exhibits a relatively stable response when the temperature change is below 20 °C, but its sensitivity rises more noticeably once ΔT reaches 20 °C. In contrast, the CNT-modified concrete (CNT_0.01%) maintains high initial sensitivity and continuously increases with further temperature elevation. A different trend is observed for the 28-day samples, as shown in Figure 14b. Before 20 °C, both CNT_0% and CNT_0.01% samples exhibited low and similar sensitivity, not exceeding 1.2 nA/°C. However, once the temperature changes over 20 °C, the influence of CNT incorporation becomes apparent, and the sensitivity of the CNT_0.01% sample begins to rise significantly with temperature. This result strongly supports the potential of CNT-enhanced concrete as a temperature-sensitive self-sensing material.

## 4. Conclusions and Discussion

This paper analyzed the electrical conductivity of mature concrete samples containing different CNT contents. The mechanical and electrical responses of CNT-enhanced multifunctional concrete samples (0 wt.%, 0.002 wt.%, 0.006 wt.%, 0.01 wt.%, 0.018 wt.%, 0.025 wt.%, and 0.05 wt.%) at different curing ages (7-day and 28-day) were investigated through three-point bending tests and thermal tests. The real-time electrical response was recorded using a LabVIEW-Picoammeter system to assess the correlation between load, temperature variations, and electrical behavior. Based on the results, the potential of CNT-reinforced concrete as a sensor was analyzed from both mechanical and thermal perspectives. The following conclusions were drawn:

(1) Electrical conductivity measurements on fully cured samples confirmed the semiconductive nature of the CNT-modified concrete. The optimal conductivity was observed at 0.006 wt.% CNTs, which reaches 2.81 × 10^−4^ S/m, indicating the formation of an effective conductive network. However, higher CNT concentrations did not lead to further improvement, likely due to agglomeration effects that disrupted conductive pathways.
(2) From the three-point bending tests, the CNT content of 0.01 wt.% was identified as the optimal balance between mechanical behavior and electrical response in 7-day samples, with a peak load of 2.6615 kN and a maximum current of 2.89 × 10^−6^ A. For 28-day samples, the mechanical behavior remained relatively stable cross all CNT contents, except for the control group. The highest load (2.9365 kN) was observed at 0.006 wt.% CNT, while the highest electrical response (8.34 × 10^−8^ A) was recorded at 0.01 wt.%.The results suggest that a CNT content of 0.006 wt.% proves optimal for mechanical property enhancement, while 0.01 wt.% is optimal for electrical property enhancement. For studies targeting both properties simultaneously, 0.01 wt.% represents the optimal dosage.
(3) The thermal response tests further support this conclusion. In 7-day samples, CNT concentrations between 0.006 wt.% and 0.018 wt.% exhibited a pronounced electrical response to thermal stimuli, with relatively small variations in peak current and time to reach the peak. The highest peak current (1.38 × 10^−8^ A) was recorded in the 0.018 wt.% sample. However, in 28-day samples, the peak current values were significantly lower, with the highest response (9.70 × 10^−9^ A) occurring at 0.01 wt.%. Furthermore, the correlation between heat flux and peak current became less evident, suggesting that hydration-related changes in microstructure affect the thermal–electrical behavior of CNT-modified concrete.
(4) The sensitivity was analyzed for both mechanical and thermal changes to further evaluate the feasibility of using CNT-reinforced concrete as a sensor material. The 7-day cured samples exhibited higher sensitivity than the 28-day ones in response to both load and temperature variations. This enhanced sensitivity can be attributed to the incomplete hydration process at early ages, which results in higher internal moisture content and a more continuous conductive network. In terms of mechanical response, the specimens demonstrated high sensitivity at low loads, indicating the potential for early-age structural damage monitoring in concrete structures. Regarding thermal response, sensitivity became higher when the temperature exceeded the safety threshold (approximately 20 °C), highlighting the promise of CNT-modified concrete for high-temperature early warning in smart building applications.
(5) Overall, our findings indicate that CNT-modified concrete with CNT concentration at 0.01 wt.%, can achieve an optimal combination of mechanical integrity and electrical sensitivity. This opens possibilities for using CNT-enhanced concrete as a self-sensing material for structural health monitoring, enabling the real-time assessment of mechanical stress and temperature variations. Future research could explore long-term durability, alternative CNT dispersion methods to enhance conductivity, and field-scale validation to further confirm the practical feasibility of CNT-based smart concrete applications in civil engineering and infrastructure maintenance.

## Figures and Tables

**Table 1:** Usage of various materials and CNT content.

CNT Content (%)	Cement (g)	Water (g)	Sand (g)	CNT (g)	Masterbatch (g)
0%	450	225	1350	0	0
0.002%	450	225	1350	0.009	0.02
0.006%	450	225	1350	0.027	0.06
0.01%	450	225	1350	0.045	0.1
0.018%	450	225	1350	0.081	0.18
0.025%	450	225	1350	0.1125	0.25
0.05%	450	225	1350	0.225	0.5

**Table 2:** Experimental parameters and results of different CNT content concrete samples.

CNT Content	R_1_ (Ω)	*ρ*_1_ (Ω·m)	*σ*_1_ (S/m)	R_2_ (Ω)	*ρ*_2_ (Ω·m)	*σ*_2_ (S/m)	*σ* (S/m)
0%	8.17 × 10^4^	6.54 × 10^3^	1.53 × 10^−4^	8.10 × 10^4^	6.84 × 10^3^	1.54 × 10^−4^	1.54 × 10^−4^
0.002%	7.49 × 10^4^	5.99 × 10^3^	1.67 × 10^−4^	7.52 × 10^4^	6.01 × 10^3^	1.66 × 10^−4^	1.67 × 10^−4^
0.006%	4.48 × 10^4^	3.59 × 10^3^	2.79 × 10^−4^	4.42 × 10^4^	3.53 × 10^3^	2.83 × 10^−4^	2.81 × 10^−4^
0.010%	5.38 × 10^4^	4.30 × 10^3^	2.32 × 10^−4^	5.46 × 10^4^	4.37 × 10^3^	2.29 × 10^−4^	2.31 × 10^−4^
0.018%	5.92 × 10^4^	4.74 × 10^3^	2.11 × 10^−4^	5.90 × 10^4^	4.72 × 10^3^	2.12 × 10^−4^	2.11 × 10^−4^
0.025%	7.49 × 10^4^	6.00 × 10^3^	1.67 × 10^−4^	7.55 × 10^4^	6.04 × 10^3^	1.66 × 10^−4^	1.66 × 10^−4^
0.05%	7.36 × 10^4^	5.89 × 10^3^	1.70 × 10^−4^	7.45 × 10^4^	5.96 × 10^3^	1.68 × 10^−4^	1.69 × 10^−4^

**Table 3:** Experimental parameters and results of different CNT content concrete samples at 7 days.

CNT Content	T_0_ (°C)	T_1_ (°C)	Heat Flux (W/m^2^)	Heating Time (s)	Max Current (nA)	Total Heat (J)
0%	23	50	200	447.00	4.51	572.16
0.002%	23	50	200	344.00	5.41	440.32
0.006%	23	50	200	544.50	13.30	696.96
0.010%	23	50	200	535.50	11.43	685.44
0.018%	23	50	200	500.00	13.84	640.00
0.025%	23	50	200	426.50	9.05	545.92
0.05%	23	50	200	345.00	3.19	441.60

**Table 4:** Experimental parameters and results of different CNT content concrete samples at 28 days.

CNT Content	T_0_ (°C)	T_1_ (°C)	Heat Flux (W/m^2^)	Heating Time (s)	Max Current (nA)	Total Heat (J)
0%	23	50	200	1083	2.88	1386.24
0.002%	23	50	200	256.5	4.46	328.32
0.006%	23	50	200	350.5	6.12	448.64
0.010%	23	50	200	546.5	9.70	699.52
0.018%	23	50	200	444	5.90	568.32
0.025%	23	50	200	700.5	6.83	896.64
0.05%	23	50	200	683.5	2.78	874.88

## Data Availability

Data is contained within the article. The original contributions presented in this study are included in the article. Further inquiries can be directed to the corresponding author(s).
